# The Effect of Stress Inoculation Training on Breastfeeding Self-Efficacy and Perceived Stress of Mothers With Low Birth Weight Infants: A Clinical Trial 

**Published:** 2018-09

**Authors:** Mohammad Mehdi Mohammadi, Roghayeh Poursaberi

**Affiliations:** 1 Students Research Committee, School of Nursing and Midwifery, Kermanshah University of Medical Sciences, Kermanshah, Iran; 2 Department of Education, Payame Noor University (PNU), Azarshar, Iran

**Keywords:** Infant, Low Birth Weight, Breast Feeding, Self Efficacy

## Abstract

**Objective:** Mothers with low birth weight infants experience more stress, which results in reduced breastfeeding self-efficacy and exclusive breastfeeding; In this regard, stress Inoculation Training (SIT) is one of the effective ways for inoculation against stress and psychological distress; Therefore, this study aimed to investigate the effect of SIT on breastfeeding self-efficacy and perceived stress of mothers with low birth weight infants.

**Materials and methods:** This clinical trial study was conducted from October to December 2017 on 100 mothers with low birth weight infants; the infants had been hospitalized in the neonatal intensive care unit (NICU) in Kermanshah, Iran. The eligible mothers were randomly divided into two groups, i.e., intervention (n = 50) and control (n = 50) groups.

**Results:** The mean score of breastfeeding self-efficacy, before SIT (33.82 ± 8.92) compared to after SIT (42.02 ± 8.83), significantly increased (p < 0.001), though no statistically significant difference was reported in the control group (p > 0.05). The mean score of perceived stress was significantly reduced after SIT (26.29 ± 6.49) compared to values before SIT (31.25 ± 5.82) (p < 0.001).

**Conclusion:** The present study showed that on the one hand, SIT can effectively increase the breastfeeding self-efficacy in mothers with low birth weight infants; on the other hand, it can reduce their perceived stress. Therefore, the need for holding in-service training courses is felt in order to train the caregiving personnel, especially nurses, in applying the SIT technique.

## Introduction

Every society relies on the health of its people to make progress. In this respect, the health of infants and children, as those who make the future of each country, assumes special importance (1). Low birth weight refers to infants whose birth weights are smaller than 2,500 grams (2); these infants are considered the population at risk, and experience many physical, mental, and psychological problems compared to normal infants (3).

Breast milk is considered the main axis of infant nutrition in such a way that breastfeeding meets all the basic needs of infants before six months of age (4). Breast milk is a biological compound that meets the nutritional balance of infant at all quantitative and qualitative levels. In this respect, the majority of national and international organizations around the world have come to a consensus on exclusive breastfeeding in the first six months of life (3, 5). The importance of breastfeeding is so much that Von Stumm et al. reported that breastfeeding is correlated with neonatal IQ growth (6). The growth and development of low birth weight infants require the use of exclusive breastfeeding. On the other hand, low birth weight infants are more susceptible to diseases; however, evidence suggests that premature and low birth weight infants are more susceptible to breastfeeding than other infants (7). The body of premature infants tries to continue its uterine growth process; to this end, these infants require the intake of more calories than normal infants. Any failure to supply this amount of calories contributes to the development of azotemia and a decrease in blood glucose levels (8).

From among the benefits of using breast milk in premature infants, one can refer to the reduction in mortality, infectious diseases, and necrotizing enterocolitis (NEC). In this respect, Cristofalo et al. (2005) showed that the premature infants who were fed formulas would be more susceptible to NEC than the infants receiving exclusive breastfeeding (9, 10).

The postpartum period is considered a sensitive period in which women are more prone to experiencing physiological distress. In mothers with low birth weight infants, this period has coincided with a critical condition that pushes the mothers to a psychological crisis. In this respect, Bener et al. showed that a large number of distress types are associated with premature infants; hence, mothers with premature infants are more prone to the development of major depression and anxiety (11). On the other hand, psychological stresses affect exclusive breastfeeding; in this connection, Ahlgvist et al. indicated marital distress can reduce the duration of the exclusive breastfeeding period (12).

Breastfeeding self-efficacy is based on Bandura’s social cognitive theory (13); it refers to the person’s belief and confidence in the ability to promote health-related behaviors, including exclusive breastfeeding (14). In this regard, Dennis et al. showed that there is a significant relationship between breastfeeding self-efficacy and the length of exclusive breastfeeding; in fact, this positive relationship is so strong that the increase in the level of breastfeeding self-efficacy can be regarded as equal to the increase in the length of exclusive breastfeeding (15).

According to Bandura’s theory, breastfeeding self-efficacy is influenced by four main sources (13):

     1- Performance Accomplishments (for example, previous breastfeeding experience)

     2- Vicarious Experiences (for example, observing other breastfeeding women)

     3- Verbal Persuasion (for example, encouragement from effective people like friends, family, and previous counselors)

     4- Physiological Responses (for example, fatigue, stress, anxiety) (14).

In this study, the main focus is on the fourth source of information, namely physiological responses; in other words, the ground for the promotion of breastfeeding self-efficacy is provided by focusing on this domain. In this regard, evidence suggests that postpartum distress has an adverse effect on breastfeeding self-efficacy and exclusive breastfeeding. On the other hand, there is a strong relationship between breastfeeding self-efficacy and exclusive breastfeeding; therefore, it is of high importance to use interventions to reduce postpartum distress and promote breastfeeding self-efficacy.

Stress Inoculation Training (SIT) is one of the effective ways for inoculation against stress and psychological distress. This inoculation technique was first introduced by Meichenbaum in 1985 (16). The SIT acts like inoculation in the medical sciences, in which the person is encouraged to produce psychological antibodies against stressful situations by being placed in a stressful position. This training is an accurate, multidimensional, and multi-faceted therapeutic intervention that does not aim to eliminate stress completely, but aims to go in for its constructive exploitation so that the clients can be encouraged to regard stressful situations not as a threat to themselves but as solvable issues. In three steps, this approach provides users with the necessary training to reduce and treat stress and stress-related problems (17). These three steps are as follows:

     1. Conceptualization: At the conceptualization stage, the focus is on the establishment of a relationship based on collaboration with clients, and helping them better understand the nature of stress and its effects on emotion and performance.

     2. Acquisition and Practice of Skills: The main emphasis is on training multiple coping skills to clients.

     3. Continuous Application and Follow-up: The person applies his/her skills to increase the ability to cope with any stress, as well as stronger stress (18).

In the present study, the researchers intend to use SIT as an intervention to reduce postpartum stress and promote breastfeeding self-efficacy in mothers with low birth weight infants. In term of previous studies, the study of Esmaeili et al. (2016) can be considered (19). In this study, the effectiveness of SIT on psychological well-being in women with diabetes mellitus type 2 was investigated. The result indicated that SIT is effective in improving psychological well-being. In another study, Cecil et al. found that SIT was not successful in changing motoric manifestations of anxiety in the classroom (20). These contradictory results highlight the importance of conducting further studies to produce convincing evidence about the effects of SIT. 

As regards the direct effect of SIT on breastfeeding self-efficacy, no study was found in the literature; therefore, the present study is completely unique in this respect.

There is a gap of knowledge with regard to the effect of SIT on breastfeeding self-efficacy and perceived stress of mothers with low birth weight infants; therefore, the present study will be conducted to determine the effect of SIT on breastfeeding self-efficacy and perceived stress in mothers with low birth weight infants.

## Materials and methods

***Design: ***The present study was a clinical trial study conducted from October to December 2017 on 100 mothers with low birth weight infants who had been hospitalized in the neonatal intensive care unit (NICU) of Kermanshah educational hospitals in Iran. These centers were governmental hospitals that had the NICU. The participants under study were randomly divided into two groups of intervention (n = 50) and control (n = 50).

***Sample size calculation: ***The sample size was calculated using the results of a pilot study. In this regard, Pocock’s table was used to estimate the sample size (21). The number of 50 sample units was ultimately estimated for each group (interventiolen and control) by means of the pilot study and by taking into account 0.01 for the first type errors (α) and 0.05 for the second error (β) and 10% for the sample unwillingness for the continued participation.

$n=\frac{S_{1}^{2}+S_{2}^{2}}{{\left(\mu_{2}-\mu_{1}\right)}^{2}}f(\alpha ,\beta )$

In this formula, S1 represents the standard deviation before the intervention, S2 shows the standard deviation after the intervention; µ_1_ is the mean before the intervention, and μ2 is the mean after the intervention. According to Pocock’s table, each of Type 1 error and Type 2 error was considered at the lowest possible level in order to reduce the level of error where the Type 1 error was considered 0.01 and the Type 2 error was considered 0.05.

According to the pilot study; S_1_=9.01 S_2_=8.64 µ_1_=34.11 µ_2_=42.02 

$n=\frac{({9.01)}^{2}+{\left(8.64\right)}^{2}}{{\left(42.02-34.11\right)}^{2}}\times 17.8$

n≅ 45 $\underset{\to }{10\mathrm{\% attrition rate}}$ ≅50 (for each group)

After the random allocation of participants in two intervention and control groups, the standard questionnaires of Cohen’s Perceived Stress Scale (PSS) and Dennis’s Breastfeeding Self Efficacy Scale (BSES) will be administered to the two groups; then, a SIT course will be conducted in the intervention group, whereas the control group will not receive any training intervention. Finally, both groups would be requested to complete PSS and BSES once more (Table 1).

**Table 1:** Pocock's table (1970)

	**Β**				
Α		0.05	0.1	0.2	0.5
	0.1	10.8	8.6	6.2	2.7
	0.05	13	10.5	7.9	3.8
	0.02	15.8	13	10	5.4
	0.01	17.8	14.9	11.7	6.6

***Inclusion criteria: ***The criteria for the inclusion of participants in the study are the mothers’ informed consent to participate in the study, the ability to read and write, the availability of the mother over telephone, single pregnancy, vaginal delivery, living with the husband, no history of mental illness, no participation in similar training programs, and the infant’s weight being lower than 2,500 grams.

***Exclusion criteria: ***The exclusion criteria are infant death, infant attachment to mechanical ventilation, the absence of a mother in training sessions (up to two sessions), and the mother’s reluctance to continue the research participation.

After examining the sample units in terms of the inclusion criteria, each of the mothers was randomly assigned to one of the two intervention and control groups (by tossing a coin). Then, the researcher distributed PSS and BSES between each of two groups (intervention and control). Thereafter, the intervention group received SIT program in the form of eight sessions. At the end, the questionnaires were distributed between the two groups once more.

***Training sessions: ***The whole training was held over eight sessions. For this purpose, the first three sessions were held in the hospital using the face-to-face approach and the next five sessions were conducted using a hybrid method via telephone and self-learning at home. The trainer was a woman specialized in psychology and stress. The first three face-to-face training sessions were conducted in one of the classes of the educational hospital. In terms of the combined teaching method (self-learning and telephonic training), a training package containing pamphlets and booklets was provided to each of the mothers in the intervention group. Prior to conducting each self-learning session, the researcher reminded them of the need to conduct the training session on that day through a telephone call to each of the sample units. During this phone call, the topics of the previous session were recapitulated by the teacher over five minutes; then, the telephonic conversation was interrupted and the mother was allowed to study the topics of self-learning. Thereafter, another telephone call was made, and the teacher fully taught the topic of that session to mothers within 15 minutes. In addition, the mothers were also given the opportunity to ask their questions about the items described in the form of self-learning (Table 2).

**Table 2:** Outline of the Training Sessions

**Training ** **sessions**	**Title of the skill being ** **trained**	**Description of training sessions**
First session	Members' acquaintance with each other and with the group leader	Review of the group goals and the expectations of individuals from attending the training sessions
Second session	Recognition of the problem as the first step (re-conceptualizing the problem)	Conceptualization of the interactive nature of stress and familiarity with various concepts and definitions of stress, as well as the unwanted effects of physiological (physical), emotional, cognitive, and behavioral stress on individuals
Third session	Mental imagery	The mental imagery technique was explained and administered.
Fourth session	Recognition, prevention, and relaxation	Familiarity with the impact of stress on fetus and the need to prevent these effects and to train relaxation
Fifth session	Cognitive strategies	In this session, a review of prior sessions and relaxation sessions was initially undertaken, and then it was explained about cognitive strategies on how bad thoughts lead to bad feelings, and bad feelings lead to bad behavior. It was emphasized on the necessity of preventing and blocking bad thoughts.
Sixth session	Time management skill, memory and ...	At this session, firstly, there were problems with how each of the techniques was handled, then the problem-solving and solvability of stressors or stress reactions was discussed.
Seventh session	Internal and directed dialogue	A personalized directed conversation aimed at helping clients to assess the conditions required for the situation, plan for future stressors, increase their "spirit" to face stressful situations, think about their own actions, and reinforce themselves to counteract.
Eighth session	Coping responses in practical situations	The training of this stage is to encourage clients to respond to coping responses in everyday practical situations and maximize the possibility of generalizing the results in their current life. At this stage, the researcher used the step-by-step mastery method where the small controllable units of stress were first presented in experimental settings and, then, were gradually transferred to real conditions. In pursuit of the above objectives, the researcher utilized a variety of techniques, including gradual training in real conditions. Behavioral exercises and mental imagery, role play, role models

***Research instruments: ***This study used Cohen’s PSS and Dennis’s BSES, which will be described here. Cohen’s PSS includes 14 statements that examine the amount of people’s thoughts and feelings. In this research, the 14-item version was used. The questionnaire items are scored on the basis of a five-point Likert scale from never (0), almost never (1), sometimes (2), often (3), and always (4). Items numbered 4, 10, and 13 are scored in reverse. The minimum score will be zero and the maximum score will be 56. The validity of this tool has been confirmed by 10 experts; experts gave their assurances as to the content validity of the questionnaire (content validity index [CVI] = 0.98). In terms of reliability, the Cronbach’s alpha coefficients of internal consistency of the scale vary in the range of 84% to 86% (18).

Another instrument used in this study is Dennis’s BSES, which included 14 questions. All questions started with the statement “I can always…”, and were designed in the form of positive sentences based on a five-point Likert scale and in accordance with Bandura’s suggestion in self-efficacy theory. Here, the range of score from one, “not at all confident” to five, “always confident,” is considered for each response; hence, the range of breastfeeding self-efficacy scores was between 14 and 70. In fact, the highest score indicates the highest breastfeeding self-efficacy and vice versa. In other words, as the scores are above the median, the person enjoys higher breastfeeding self-efficacy and vice versa; when the person’s score was lower, the person has a low level of self-efficacy (14). The validity was confirmed with the help of 10 experts with the evaluation registering acceptable face and content validity (CVI = 0.98). In terms of reliability, the previous studies have confirmed the reliability of the questionnaire using Cronbach’s alpha coefficient (higher than 0.7), which is acceptable (22, 23).

**Figure 1:**
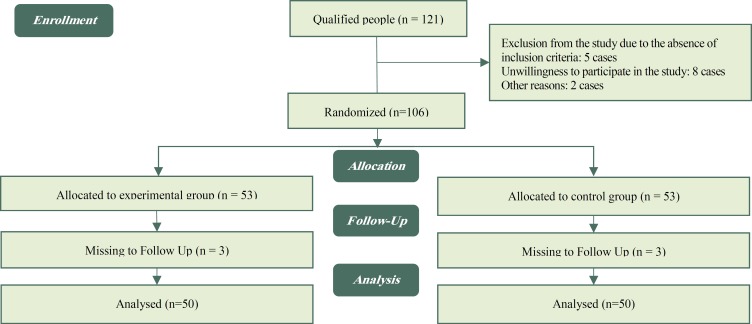
Consort Flow Diagram of the Study

***Data collection procedure: ***For data collection, the necessary permits were obtained from the Research and Technology Department and the Ethics Committee of Kermanshah University of Medical Sciences. Then the researchers, along with a female psychologist, referred to each of the educational hospitals in Kermanshah that held a NICU. After achieving the necessary coordination with the head of each department, they identified the mothers with low birth weight infants who enjoyed the inclusion criteria. For this purpose, they referred to each of the mothers and the research objectives were explained to each of them, and their written and informed consent was obtained. Accordingly, at first, 121 sample units were recognized as eligible to enter the study; however, because of the absence of inclusion criteria, unwillingness to participate in the study, and other reasons, 15 people were initially excluded and the remaining 106 were randomly assigned to the intervention and control groups through coin tossing (line or milk). After the random assignment, and during the process of following up the samples, three participants were excluded from each group. Finally, the data were analyzed with 50 subjects in each group (Figure 1).

**Table 3:** Comparison of demographic variables between the intervention and control groups

**Variable**		**Group**		**P-value**
		**Intervention**	**Control**	
Infant gender (frequency)	Male	27	25	0.841
	Female	23	25	
Type of delivery (frequency)	Cesarean section	30	27	0.686
	Vaginal delivery	20	23	
Economic problems of treatment costs (frequency)	Low	12	15	0.766
	Moderate	24	21	
	High	14	14	
Birth weight (g)		2116 ± 259	2132 ± 241	0.751
Mother's age (mean)		28.41 ± 3.38	29.64 ± 3.36	0.723
Mother's education level (frequency)	Illiterate	5	6	0.932
	Primary school	11	12	
	Diploma	17	13	
	Associate's	7	7	
	Bachelor and above	10	12	

***Ethical considerations: ***For data collection, while the objectives of the study were explained to each of the subjects, they were assured that their information would be kept confidential and they could resign participation in the study during the study whenever they wished (Ethical code: IR.KUMS.REC.1396.343). 

***Data management and analysis: ***The data were analyzed using SPSS Version 16. Descriptive statistics (frequency, percentage, mean, and standard deviation) and inferential statistics methods (including Chi-square test, independent t-test, and paired t-test) were used. In this respect, the data were normalized using descriptive evidence and the Kolmogorov–Smirnov test; the distributions of all variables were normal. Subsequently, the demographic variables were analyzed using the Chi-square test. In addition, the independent t-test was used for the comparison of the two groups and the paired t-test was used to compare the two dependent groups. For the analysis of data, we used the Per Protocol (PP) method. All protocol deviations considered as major will lead to the exclusion of patients from the analysis.

## Results

The mean and standard deviation of the age in the intervention and control groups equaled 28.41 ± 3.38 and 29.44 ± 3.36 respectively. In this respect, the independent t-test showed no significant difference (p > 0.05). On the other hand, the intervention and control groups did not show any significant difference in the comparison of demographic variables (p > 0.05) (Table 3).

Table 4 shows the mean scores of breastfeeding self-efficacy in both intervention and control groups. As shown in the table, the results of the t-test show that there is a significant difference between the intervention group before and after the intervention in terms of breastfeeding self-efficacy (p < 0.001). In this respect, the mean score of breastfeeding self-efficacy in the intervention group has increased (p < 0.001) while the control group did not report any significant statistical difference in terms of breastfeeding self-efficacy with the passage of time (p > 0.05). On the other hand, an independent t-test showed that there was a significant difference between the mean scores of intervention and control groups (p < 0.001) (Table 4).

In mothers with low birth weight infants, the mean score of perceived stress significantly decreased after the training sessions (p < 0.001). On the other hand, the independent t-test showed a significantly higher rate of perceived stress in the intervention group than that in the control group (p < 0.001) (Table 5).

**Table 4:** Mean and standard deviation of breastfeeding self-efficacy in pre-test and post-test in both intervention and control groups

**Variable**	**Groups**	**Before intervention**	**After intervention**	**Paired t-test**
		**Mean ± SD**	**Mean ± SD**	
Breastfeeding self-efficacy	Experimental	33.82±8.92	42.02 ± 8.83	P = 0.0001
	Control	33.31±8.94	33.77 ± 9.41	P = 0.523
	Independent t test	P = 0.785	P = 0.0001	

**Table 5:** Mean and standard deviation of perceived stress in pre-test and post-test in both intervention and control groups

**Variable**	**Groups**	**Before intervention**	**After intervention**	**Paired t-test**
		**Mean ± SD**	**Mean ± SD**	
Perceived stress	Experimental	31.25 ± 5.82	26.29 ± 6.49	P = 0.0001
	Control	32.55 ± 7.09	32.56 ± 7.05	P = 0.844
	Independent t test	P = 0.319	P = 0.001	

## Discussion

The present study showed that SIT can increase the breastfeeding self-efficacy in mothers with premature infants and reduce their perceived stress.

With regard to the positive effect of SIT on the promotion of breastfeeding self-efficacy, it seems that the SIT is considered a psychological vaccination that emphasizes the physiological responses like stress and anxiety, and provides the grounds for the promotion of breastfeeding self-efficacy in mothers with premature infants by reducing these responses (14, 17). In this regard, Karbandi et al. showed that the employment of the relaxation training program can significantly improve breastfeeding self-efficacy in mothers with premature infants. The reasons given by Karbandi et al. in explaining this claim were indicative of the fact that one of the factors influencing the creation of an imbalance in breastfeeding self-efficacy comprises physiological responses, including stress and anxiety. Since the relaxation method acts as a proven intervention in reducing stress, this intervention can also promote breastfeeding self-efficacy (24). Based on Karbandi’s explanation, this finding was supported in this study viz. reduced stress can enhance breastfeeding self-efficacy in some way and, thereby, a new psychological intervention was used to balance both the stress and breastfeeding self-efficacy. The intervention used in this study is much more efficient than a simple relaxation training program. In addition to the individual’s empowerment to employ stress relief tools such as relaxation, SIT attempts to somehow immunize the individual against stress with an emphasis on cognitive strategies. This immunization resembles the one that is used to inject a vaccine in the fight against physical illness with the difference that antibodies made here are psychological antibodies and that the person here is exposed to low doses of stress and can thereby cope with stress in real conditions (17). The review of the related literature did not result in the availability of any studies that have assessed the direct effect of SIT on breastfeeding self-efficacy. The present study is a novel research in this field that introduces a strong psychological strategy to enhance breastfeeding self-efficacy.

Another finding of the present study was that SIT can reduce the perceived stress of mothers with low birth weight infants. Postpartum stress provides an area of imbalance in mothers with premature infants; these mothers, on the one hand, are prone to postpartum depression and anxiety; on the other hand, the stress caused by the birth of low weight infant orients them to an imbalanced state more than ever (11). However, the findings suggest that stress leads to impairment in exclusive breastfeeding self-efficacy (12, 14). In other words, stress can be considered the main link in the reduction of exclusive breastfeeding, which can lead to an impairment of exclusive breastfeeding, both directly and indirectly, through its effect on breastfeeding self-efficacy. Consistent with this study, Khorsandi et al. had also examined the effect of SIT on the decrease in pregnant mothers’ stress. These mothers had still not experienced a postpartum period where mothers are more susceptible to anxiety and stress, and this stress will increase dramatically if the newborn is premature or has a low weight (11, 25). The present study focused on reducing postpartum stress; it was different from the study conducted by Khorsandi et al. Hasanzadeh et al. also assessed the effect of SIT on pregnant women with a history of infertility and showed that this intervention can reduce perceived stress in these pregnant mothers (18). However, it is necessary to pay more attention to the use of SIT in the postpartum period and in mothers with premature infants.

One of the limitations of this study is the impact of some social variables on the effectiveness of breastfeeding self-efficacy in mothers with low birth weight infants. In the present study, the control of some of these variables was beyond the researcher’s ability. However, an attempt was made to reduce the impact of these unwanted variables by means of the homogenization of the two groups as well as by random assignment. With regard to the studies which the researchers intend to carry out in future, it is suggested that the length of the breastfeeding period be measured in addition to the evaluation of breastfeeding self-efficacy. 

Nevertheless, this study has a certain strong point. The study is unique in terms of examining the effect of SIT on breastfeeding self-efficacy in mothers with low birth weight infants.

## Conclusion

The present study showed that SIT can effectively increase breastfeeding self-efficacy in mothers with low birth weight infants and, on the other hand, can also reduce their perceived stress. These two important results can provide the status for the higher mastery of the mothers with premature infants over their available condition. In this way, the reduction in mothers’ stress and the increase of their breastfeeding self-efficacy lead to the better nutritional status of the low birth weight infant, since the mother with a low weight infant should be dominant over the conditions more than other mothers and should attempt to meet the nutritional needs of her baby. According to the results of this study, there is a need for the implementation of SIT intervention by the treatment personnel, especially nurses. The emphasis on the importance of learning SIT interventions for nurses is that nurses have an important caring behaviors toward patients (26). In case of the nurses’ dominance over SIT technique and its training to the patients, it is possible to provide a basis for the mastery of mothers with a premature baby over the critical conditions. Hence the need for in-service training courses to teach SIT, especially to nurses working in neonatal intensive care units.
